# Outbreaks of acute gastroenteritis associated with a re-emerging GII.P16-GII.2 norovirus in the spring of 2017 in Jiangsu, China

**DOI:** 10.1371/journal.pone.0186090

**Published:** 2017-12-28

**Authors:** Jian-Guang Fu, Chao Shi, Cheng Xu, Qin Lin, Jun Zhang, Qian-Hua Yi, Jun Zhang, Chang-Jun Bao, Xiang Huo, Ye-Fei Zhu, Jing Ai, Zheng Xing

**Affiliations:** 1 Medical School and Jiangsu Key Laboratory of Molecular Medicine, Nanjing University, Nanjing, China; 2 Key Lab of Enteric Pathogenic Microbiology, Ministry of Health, Jiangsu Provincial Center for Disease Control and Prevention, Nanjing, China; 3 Wuxi Center for Disease Control and Prevention, Wuxi, China; 4 The Second Affiliated Hospital of Nanjing Medical University, Nanjing, China; 5 Changzhou Center for Disease Control and Prevention, Changzhou, China; 6 Yangzhou Center for Disease Control and Prevention, Yangzhou, China; 7 Taizhou Center for Disease Control and Prevention, Taizhou, China; 8 Suzhou Center for Disease Control and Prevention, Suzhou, China; 9 College of Veterinary Medicine, University of Minnesota at Twin Cities, Saint Paul, Minnesota, United States of America; University of Hong Kong, HONG KONG

## Abstract

A total of 64 acute gastroenteritis outbreaks with 2,953 patients starting in December of 2016 and occurring mostly in the late spring of 2017 were reported in Jiangsu, China. A recombinant GII.P16-GII.2 norovirus variant was associated with 47 outbreaks (73.4%) for the gastroenteritis epidemic, predominantly occurring in February and March of 2017. Sequence analysis of the RNA-dependent RNA polymerase (RdRp) and capsid protein of the viral isolates from these outbreaks confirmed that this GII.P16-GII.2 strain was the GII.P16-GII.2 variant with the intergenotypic recombination, identified in Taiwan, Hong Kong, and other cities in China in 2016. This GII.P16-GII.2 recombinant variant appeared to a re-emerging strain, firstly identified in 2011–2012 from Japan and USA but might be independently originated from other GII.P16-GII.2 variants for sporadic and outbreaks of gastroenteritis in Japan and China before 2016. Further identification of unique amino acid mutations in both VP1 and RdRp of NoV strain as shown in this report may provide insight in explaining its structural and antigenic changes, potentially critical for the variant recombinant to gain its predominance in causing regional and worldwide epidemics.

## Introduction

Norovirus (NoV) is regarded as the most common cause of acute gastroenteritis worldwide [1]. NoV infection has been associated with 18% of all cases of acute gastroenteritis worldwide and is responsible for almost half of foodborne gastroenteritis outbreaks and 75%–90% of non-bacterial gastroenteritis outbreaks [1, 2].

Among the six known NoV genogroups (GI–GVI), only genogroup I, II, and IV NoVs infect humans. GII NoVs have been subdivided into 27 genotypes based on the RdRp gene and 22 genotypes on the capsid gene [3]. Despite the extensive genetic diversities among NoVs, GII.4 has been dominant globally for two decades. Only in recent years, a newly emerged GII.17 variant surpassed GII.4 and has become the dominant strain in causing NoV outbreaks during the 2014–2015 epidemic season in Asian countries [4, 5].

NoV outbreaks increased significantly in the winter of 2016 over the previous seasons in China and the outbreaks were predominantly associated with a recombinant variant of GII.P16-GII.2 [6, 7]. The same recombinant NoV was also believed to cause both sporadic and outbreaks concurrently in the winter of 2016 in Germany and France [8, 9]. This GII.P16-GII.2 appeared to emerge in late summer in China, firstly identified in a sample collected in August [6]. As shown in recent reports that likely the same virus caused sporadic infection and outbreaks and was detected in Guangdong [7] and Taiwan [10] in September of 2016. Dozens of the outbreaks associated with this recombinant variant had been reported by the end of December 2016 in mainland China [6, 7].

We did not, however, observe a drastic increase of gastroenteritis outbreaks in the winter of 2016 in Jiangsu province, China, compared to those in the previous years. In this report, we show that among seven outbreaks caused by NoVs in December, only one was associated with this GII.P16-GII.2 variant. Suddenly increased outbreaks of acute gastroenteritis in Jiangsu started in February and continued in March of 2017. Our data confirmed that the spring epidemic of viral gastroenteritis was caused by this GII.P16-GII.2variant, a recombinant of GII RdRp and GII VP1. We sequenced and analyzed the viral genome, including a 3.5 kb genomic fragment and the complete sequences, and our phylogenetic analysis indicated that this GII.P16-GII.2 was closely related to the recombinant variants firstly identified in 2011–2011 in Japan and USA in both RdRp and VP1 regions but differed from other GII.P16-GII.2 strains responsible for sporadic and outbreaks in Japan, and China before 2016, including the first GII.P16-GII.2 recombinant reported in 2008–2010 in Osaka, Japan [11]. Mutations in the RdRp region and VP1 protein P2 domain of this virus were further characterized in order to understand how the re-emerging GII.P16-GII.2 recombinant variant became capable of breaking herd immunity and gaining its predominance in humans starting in the season of 2016–2017.

## Materials and methods

NoV outbreak data were obtained from two surveillance systems. The first was the Emergent Public Health Event Information Management System, in which an acute gastroenteritis outbreak was defined as ≥20 cases with symptoms including vomiting and/or diarrhoea within one week. The second was the NoV outbreak surveillance system in Jiangsu province, in which an outbreak was defined as ≥5 cases with symptoms of vomiting and/or diarrhoea within three days. Data are available for public access from either Patric, a public data repository, at https://www.patricbrc.org/workspace/zxing@patricbrc.org/home or the Jiangsu Provincial Center for Disease Control and Prevention Institutional Data Access/Ethics Committee for researchers and part of data sets were submitted as the Supporting information (S1 and S2 Tables). NoV positive specimens of each outbreak were genotyped based on the ORF1 (RdRp) and ORF2 (capsid VP1) regions as previously described [4]. The genotypes were determined by using the Norovirus Automated Genotyping Tool (http://www.rivm.nl/mpf/norovirus/typingtool). To characterize the temporal and spatial distribution of the outbreaks, hierarchical mapping was carried out with ArcGIS software (version 10.0; ESRI, Redlands, CA).

To identify potential antigenic mutations, the 3.5 kb genomic fragments of twelve viral strains and the complete genomic sequences of three viral strains were amplified with polymerase chain reaction (PCR). The 3.5 kb genomic fragment contains a complete capsid sequence and partial RdRp sequence. In detail, extracted viral RNA was reverse transcribed to cDNA with a VN3T20 primer by using the Superscript III cDNA synthesis kit (Invitrogen, CA, US). Five fragments were amplified, which included three overlapping fragments for ORF1 using three pairs of designed primers (Table 1), one fragment for ORF1-ORF2 overlap using GP-F and G2SKR primers, and one fragment for ORF2 and ORF3 using COG-2F and VN3T20 primers as previously described [4]. Subsequently, the resulting NoV sequences were analyzed using CLUSTAL X (Version 1.83), subjected to phylogenetic analysis performed with MEGA v.7.0 [12]. Sequences were deposited in the GenBank under the accession numbers KY806290 to KY806301, and MF1676750 to MF167652.

**Table 1:** List of primers used in the study.

Primers	Sequences (5′-3′)	Polarity	Region^a^
GY-F	ATG AAG ATG GCS TCT AAC GAC GCT	+	1–24
GY-R	TTC TTY GGG AAR AAC CAY TTC ATR AC	-	1156–1181
GE-F	GTB TWY TGG ACH CCS CCW GAT GTS TCY	+	925–951
GE-R	TGT TBC CRT TCT TRT CRA ANC CRC CYT G	-	1960–1987
GS-F	CCD GGY CAR CCH GAY ATG TGG AAR RA	+	1888–1913
GS-R	TDG GDC CAT CYT CAT TCA TRT TCA TVC C	-	4213–4240
GP-F	GCW GAY CAR GCH TCM AAR GC	+	3982–4001
G2SKR	CCR CCN GCA TRH CCR TTR TAC AT	-	5357–5379
COG-2F	CAR GAR BCN ATG TTY AGR TGG ATG AG	+	4993–5018
VN3T20	GAG TGA CCG CGG CCG CTT TTT TTTTTTTTTTTTTTT	-	Poly A

^a^ Location on the NoV Akita8 genome (GenBank accession No. LC145786)

To determine the recombination nucleotide (nt) breakpoint and similarity to the putative parental NoV strains, the complete genome sequences of the viral strains in this study were analyzed with the reference strains obtained from GenBank by using a Simplot software v.3.5.1[13]. The bootstrap values were plotted for a window of 200 nt, moving in increments of 20 nt along the alignment.

## Results

### NoV outbreaks in the season of 2016–2017 in Jiangsu

**Fig 1:**
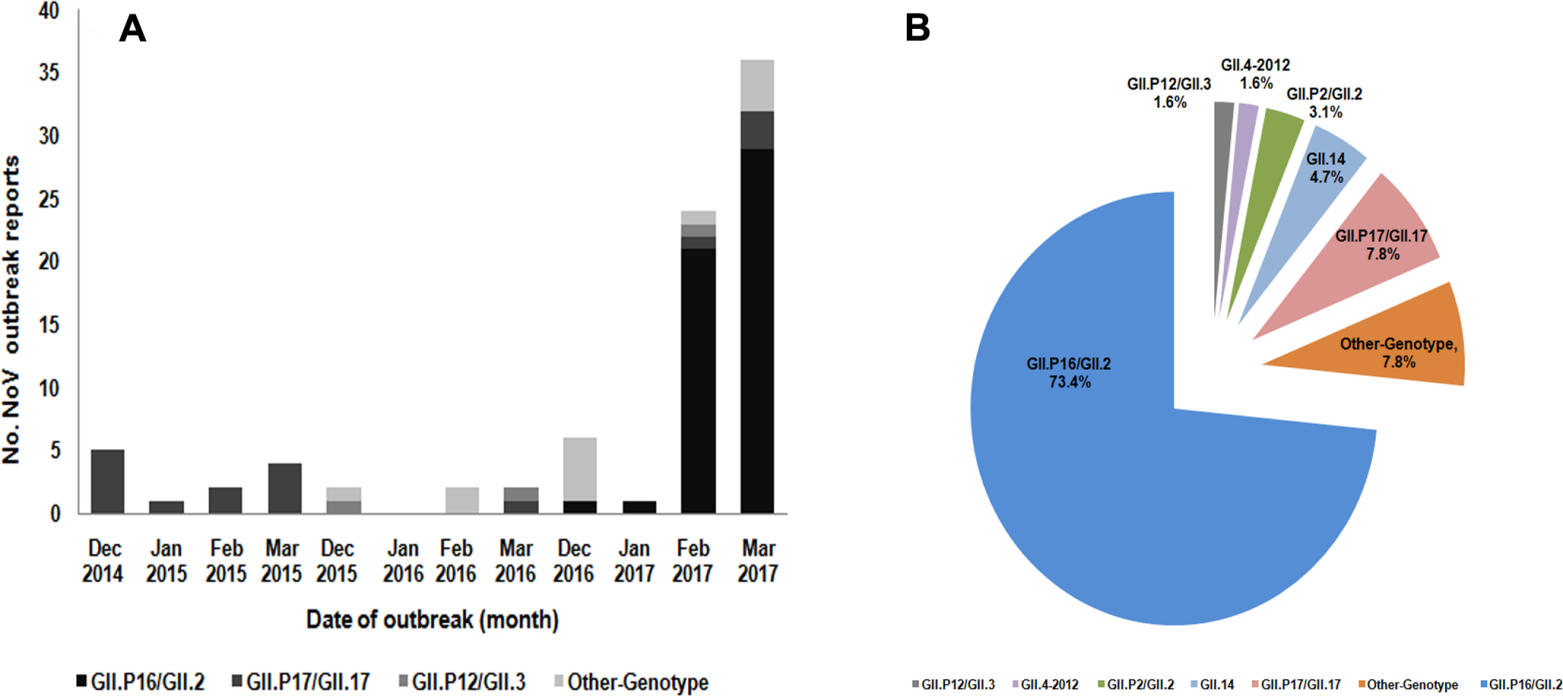
Norovirus outbreaks in Jiangsu, China. (**A**) Laboratory confirmed number of monthly NoV outbreaks occurring in Jiangsu province, China from December 2016 through March 2017. (**B**) Distribution of norovirus genotypes detected in Jiangsu from December 2016 through March 2017.

From December 2016 to March 2017, a total of 64 NoV outbreaks were reported with 2,953 clinical cases in 10 of 13 cities of Jiangsu province (Fig 1A & S1 Table). Among the outbreaks, 47 (73.4%) were associated with the emerging recombinant variant GII.P16-GII.2 (Fig 1B), which has rarely been reported in China before 2016. This genotype was firstly detected in one outbreak that happened on December23, 2016 in Jiangsu. There was only one outbreak associated with this recombinant as well in January of 2017. Drastic increase of outbreaks, however, occurred in February when schools resumed after the weeks-long winter break and the outbreaks spread rapidly throughout the province, becoming predominant in February and March 2017 (Fig 1A).

The breakup of the causes for the gastroenteritis outbreaks was shown in Fig 1B. In addition to the emerging variant GII.P16-GII.2, a fraction of the outbreaks were caused by other genotypes of noroviruses since December of 2016, which included GI.6 (2, 3.1%), GI.7 (1, 1.6%), GII.Pg-GII.1 (1, 1.6%), GII.P2-GII.2 (2, 3.1%), GII.4-Sydney2012 (1, 1.6%), GII.P12-GII.3 (2, 3.1%), GII.14 (3, 4.7%), and GII.17 (5, 7.8%) in the winter season of 2016–2017. Of the 64 outbreaks, 46 (71.9%) occurred in schools and colleges, 3 (4.7%) in factories, and 15 (23.4%) in kindergartens. Compared with the previous NoV strains, the GII.P16-GII.2 appeared highly epidemic, demonstrated with a drastic rise of outbreaks and most cases reported in February and March of 2017 (Fig 1A).

**Fig 2:**
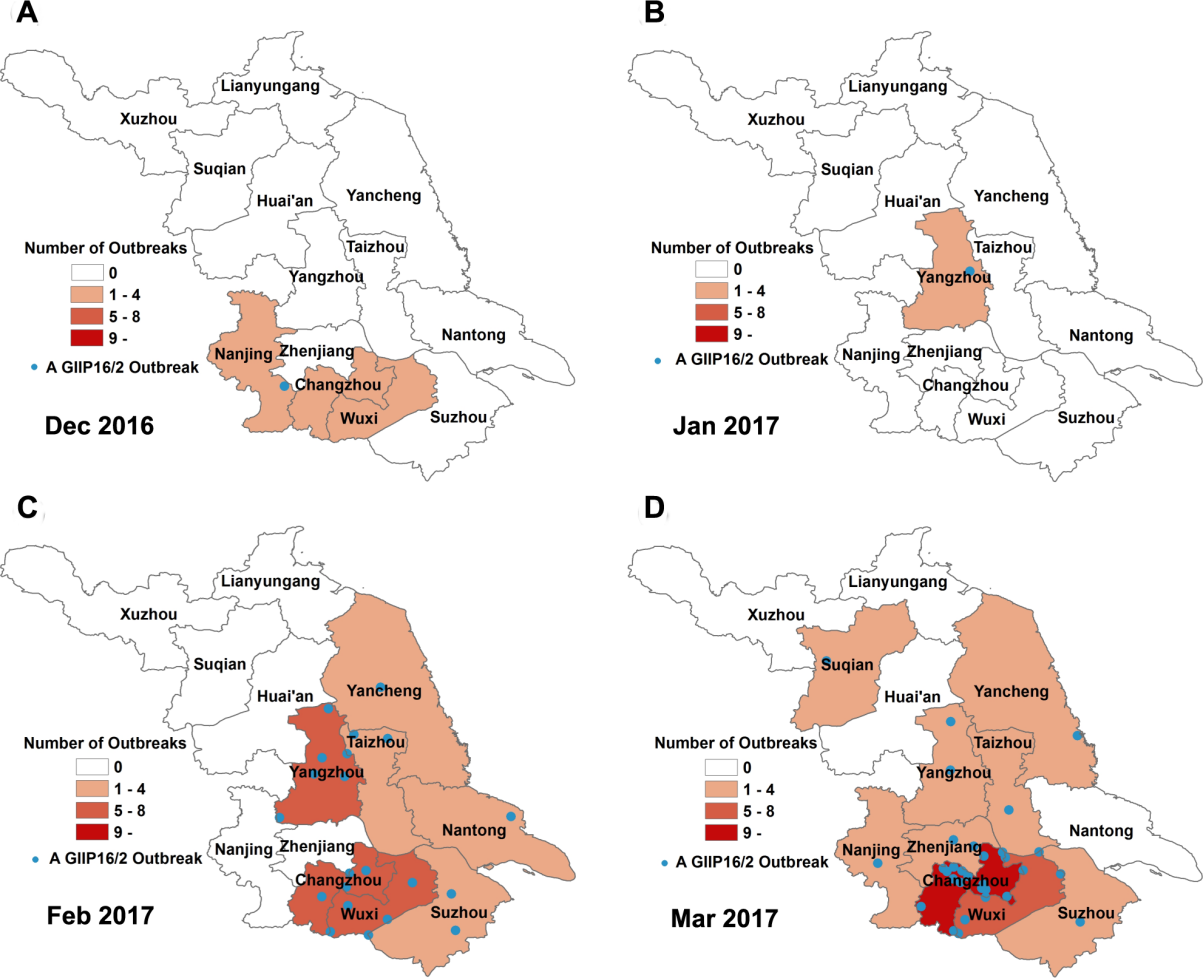
Spatial distribution of NoV outbreaks in Jiangsu from December 2016 through March 2017. The outbreaks were shown by month and geographic locations. The number of the outbreaks was reflected with darkness of color. Blue dots indicate a NoV GII.P16-GII.2 outbreak.

Spatially the outbreak with GII.P16-GII.2 started in the southern city, Nanjing, of the province in December; the only one that was reported in January occurred in Yangzhou in the central (Fig 2A & 2B; S2 Table). Outbreaks intensified in February and continued in the southern and eastern cities and later spread to Suqian, a northern city in the province in March (Fig 2C & 2D; S2 Table).

### Phylogenetic analysis of the NoV genotypes

**Fig 3:**
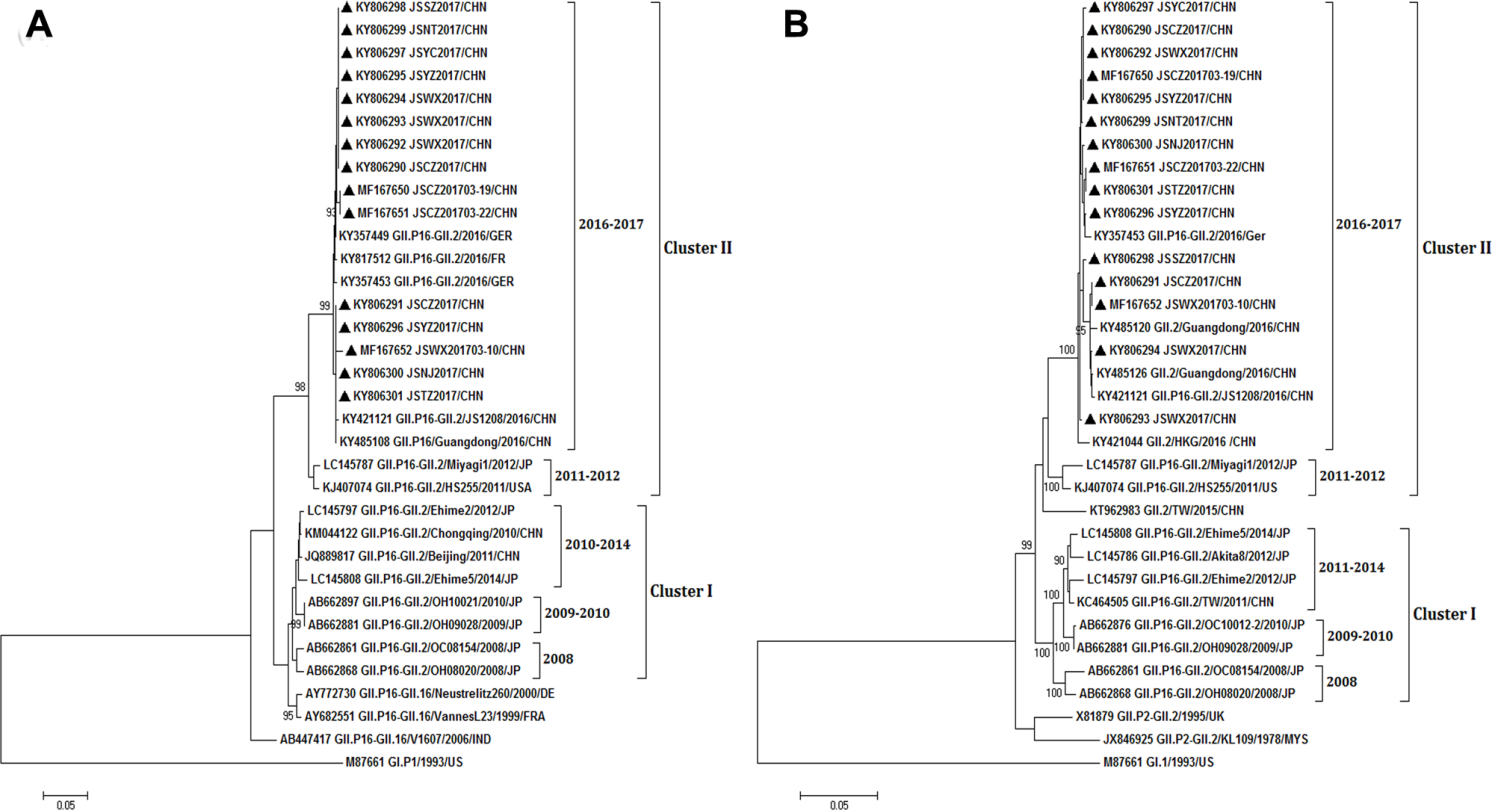
Phylogenetic analyses of the NoV GII.P16-GII.2 sequences based on partial RdRp and full length capsid regions (VP1). (**A**) Phylogenetic tree of a 1061 bp region of RdRp. (**B**) Phylogenetic tree of a 1629bp region ofVP1. The trees were constructed using the Neighbor-Joining analysis and the evolutionary distances were computed using the Kimura 2-parameterþG method available in MEGA v7.0. Bootstrap values (>90%) are shown as percentages derived from 1,000 samplings at the nodes of the tree. The scale bars represent the number of nucleotide substitutions per site. The norovirus strains reported in this study were marked with solid black triangles.

Based on the phylogenetic analysis, RdRp and VP1 gene sequences of noroviruses were grouped into two major clusters (I and II). The larger clusters were further defined into lineages, which were characterized by conserved amino acid substitutions. Sequences in cluster I were split into three lineages (2008, 2009–2010, 2010/2011-2014), and in cluster II were divided into lineages 2011–2012 and 2016–2017. The re-emerging variant GII.P16-GII.2 belonged to a single lineage 2016–2017 for RdRp region including strains from China, Germany and France of 2016–2017 season (Fig 3A) and for the VP1 region including isolates from China and Germany of 2016–2017 (Fig 3B), closely related to the lineage 2011–2012 including the strains from Japan and USA for both RdRp and VP1 region. This cluster II was separate from the cluster I with recombinant strains isolated before 2016, which included those in 2010–2014 from Japan and China and probably the earliest ones in 2008–2010 in Japan (Fig 3A and 3B). This suggests that the GII.P16-GII.2 in this report could be a re-emerging variant evolved from the isolates in 2011–2012 from Japan (LC145787) and USA (KJ407074) but may be independently originated from the other GII.P16-GII.2 strains that caused sporadic and outbreaks in Japan (LC145797) and China (JQ889817) before 2016, including the earliest recombinant GII.P16-GII.2 reported in 2008–2010 in Japan (AB662861).

**Fig 4:**
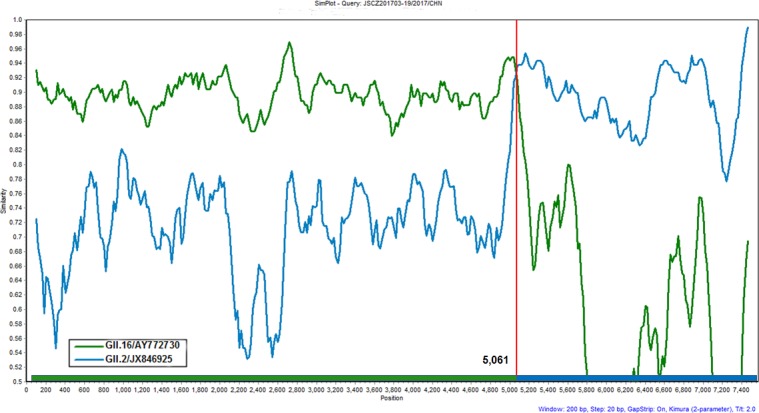
SimPlot analysis of the complete genomic sequence of GII.P16/GII.2 for recombination. SimPlot was constructed using a Simplot software version 3.5 with a slide window width of 200 bp and a step size of 20 bp. At each position of the window, the query sequence was compared to each of the reference strains (GII.16/AY772730 and GII.2/JX846925). The X-axis indicates the nucleotide positions in the multiple alignments of the NoV sequences; and the Y-axis indicates nucleotide identities (%) between the query sequence (JSCZ201703-19) and the NoV reference strains.

The complete genome sequence of a representative isolate from this study, JSCZ201703-19 (MF1676750), was further analyzed by SimPlot to determine the presence of NoV genomic recombination. As shown in Fig 4, JSCZ201703-19 shared a high level of identity in nucleotide sequences in ORF1 with the GII.16/AY772730 strain, but in ORF2 and ORF3 with the GII.2/JX846925 strain. The breakpoint of recombination was located near the ORF1/2 overlap region at nt position 5061 where the two parental strains shared the same identity and likely recombined (Fig 4).

### Phylogenetic analysis of RdRp genes

**Fig 5:**
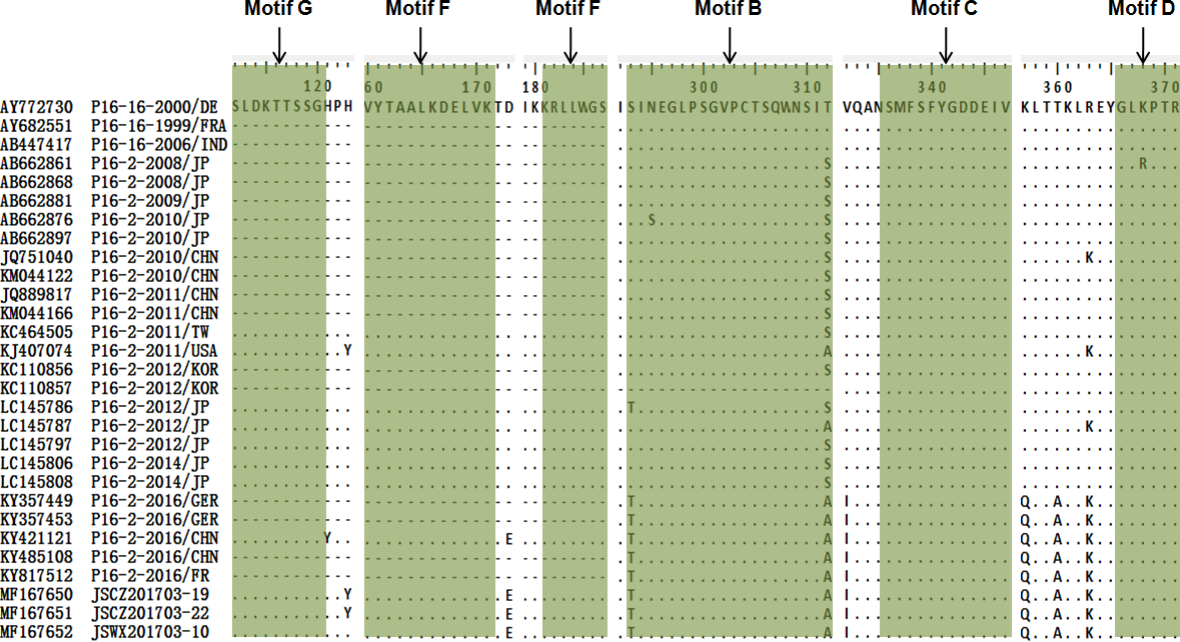
Amino acid differences between 29 NoV strains in the RdRp region. Green regions indicate the conserved residues organized into motifs involved in enzymatic activities. Dashes indicate lack sequence information. P16-16 represents GII.P16-GII.16 strain and P16-2 represents GII.P16-GII.2 strain.

According to the structure, RdRp is composed of the fingers, thumb, and palm subdomains. There are highly conserved residues organized into motifs A through G [14]. To identify specific mutations in RdRp, an alignment of amino acid sequences of the 29 GII.P16 RdRps was performed (Fig 5). Sequence data showed that there were no amino acid mutations in motifs A through G and RNA binding site (data not shown). However, four amino acid variations (D173E, V332I, K357Q, T360A) of the lineage 2016–2017 were identified, which are unique to the other lineages. The D173E substitution was located in the fingers subdomain of the motif F and the remaining three mutations located in the palm subdomain of the motifs C and D (Fig 5).

### Phylogenetic analysis of VP1 genes

**Fig 6:**
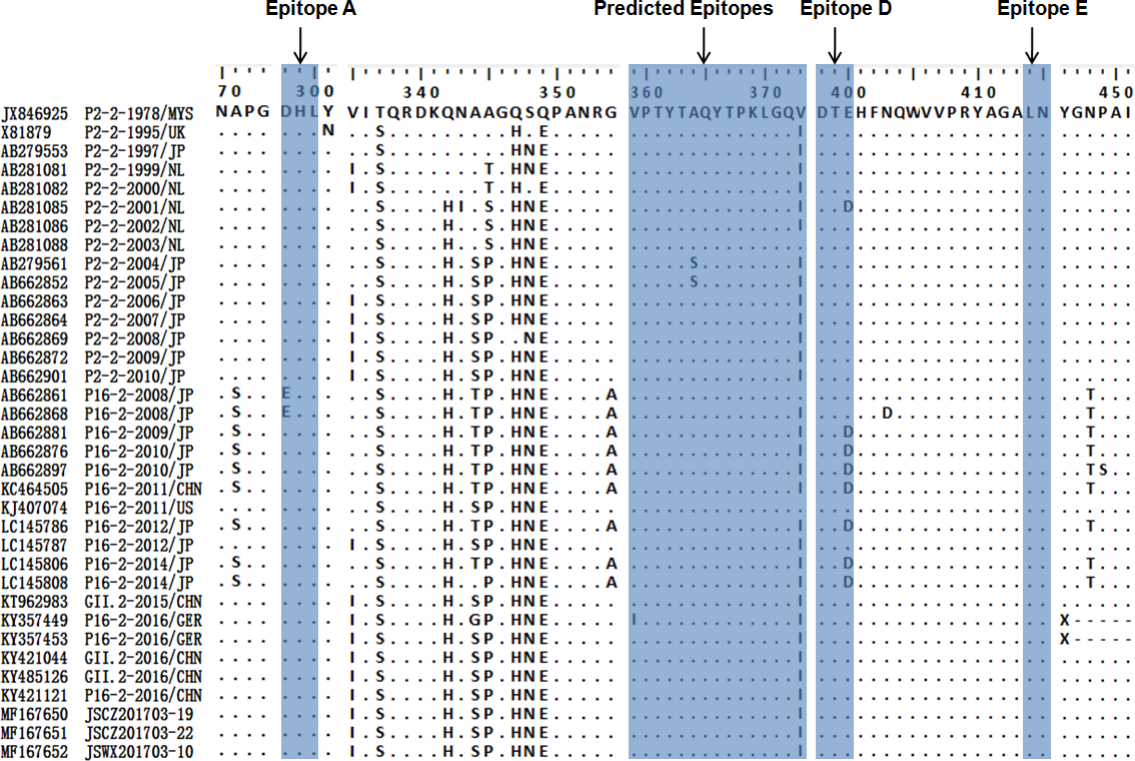
Amino acid differences between 35NoV strains in the VP1 region. Blue regions indicate some conserved amino acid substitutions which align with residues of the predicted antibody binding regions in GII.4 (epitope A, D, E) and the predicted epitopes in GII.2. P2-2 represents GII.P2-GII.2 strain and P16-2 represents GII.P16-GII.2 strain.

To identify structures of evolutionary importance, 35complete capsid protein VP1 sequences of NoV GII.2 from 1975 to 2016 were aligned, including 15 GII.P2-GII.2, 14 GII.P16-GII.2, three GII.2 (lack of the RdRp sequence), and three GII.P16-GII.2 sequences obtained in this study (Fig 6). Sequence data showed that GII.P16-GII.2 strains in the 2016/17 season were of a different genetic cluster from that of other GII.2 strains. There were no mutations in the amino acids of the predicted epitopes, but a unique amino acid change was detected at position 400, which is part of the epitope D [15, 16]. Furthermore, there were five (A71S, V335I, T344S, A354G, and T448S) mutations in the P subdomain of the VP1 protein. These mutated amino acids were more consistent with the previous GII.P2-GII.2 variants than the previous GII.P16-GII.2 variants.

## Discussion

NoV has been the leading cause of acute gastroenteritis and the GII.4 genotype has been the main cause for most outbreaks caused by NoV in the past twenty years. As the first non-GII.4 epidemic variant, GII.17 caused an increasing number of outbreaks during the 2014/15 winter season in Japan and China [4, 5]. The GII.P16-GII.2 recombinant variant emerged and reported firstly in 2008, Osaka, Japan [11]. Over the past ten years, the GII.P16-GII.2 variant was usually observed in sporadic infections with low detection rate. Increased detection of GII.P16-GII.2 as the cause of sporadic cases as well as outbreaks in Japan, USA, Germany, and China has been reported in past several years [9, 17]. The GII.P16-GII.2 variant reported in this study as well as those in China in 2016 has caused outbreaks increased by several fold in terms of incidences, compared to those caused by the GII.17 variant. Rapid increase of acute gastroenteritis outbreaks caused by this GII.P16-GII.2 variant started in the late summer and spread quickly in the winter of 2016 in many cities, China [6, 7, 10]. Our report showed that this re-emerging recombinant variant firstly appeared in December of 2016 in Jiangsu, an eastern province of China, and spread extensively in February and caused rapidly increased outbreaks in the late spring of 2017.

Recombination and point mutation are both driving forces significant in RNA viral evolution. Most recombination in NoV occurs at a single location, the ORF1-ORF2 overlap, which is composed of parts of the RdRp and VP1 regions [18]. The re-emerging recombinant variant as reported in this study as well as those recently reported in China belongs to an intergenotypic recombination. Based on the amino acid substitutions analysis of RdRp and VP1 genes, this variant showed unique mutations in both regions, especially in the VP1 gene P subdomain, which may be related to its evasion of pre-existing herd immunity and gaining eminence in spreading in 2016–2017 [11].

RdRp of NoV is a key enzyme responsible for transcription and replication of the viral genome, which plays an important role in pathogenesis and epidemiology [14, 19, 20]. Mutations of RdRp may affect its enzymatic efficacy and viral replicative efficiency, eventually leading to emergence of new variants and epidemics. Previous epidemics caused by the GII.17 NoVs were driven by the variants with a new RdRp genotype. This re-emerging recombinant variant, belonging to a new lineage, has multiple unique mutations in the RdRp region, which could be critical for causing the outbreaks with such a magnitude in Jiangsu as well as in many other cities in China.

In conclusion, a re-emerging GII.P16-GII.2 variant was detected in the winter season of 2016 to 2017 and caused outbreaks mostly in the late spring of 2017 in many cities in Jiangsu Province, China. Molecular and phylogenetic analyses showed that the re-emerging variant had unique mutations, including the intergenotypic recombination, in the RdRp and VP1 regions. It is possible that this recombinant variant, bearing substitutions in regions of the viral genome important in affecting its replicative efficiency, as demonstrated by previous GII.4 variants, may have evolved to obtain novel capacity of evading host immunity. Continuous monitoring this re-emerging variant epidemically in both outbreaks and sporadic cases is essential to further understand the importance of NoV evolution and its significance in public health.

## Supporting information

S1 Table Data file for Norovirus outbreaks in Jiangsu, China. (XLS)

S2 Table Data for Spatial distribution of NoV outbreaks in Jiangsu from December 2016 through March 2017. (XLS)
